# How do behavioral public policy experts see the role of complex systems perspectives? An expert interview study

**DOI:** 10.1093/tbm/ibae024

**Published:** 2024-05-22

**Authors:** Sarmite Puukko, Matti T J Heino, Katri Kostamo, Kaisa Saurio, Falko F Sniehotta, Nelli Hankonen

**Affiliations:** The Unit of Social Research, Faculty of Social Sciences, Tampere University, Tampere, Finland; The Unit of Social Research, Faculty of Social Sciences, Tampere University, Tampere, Finland; The Unit of Social Research, Faculty of Social Sciences, Tampere University, Tampere, Finland; The Unit of Social Research, Faculty of Social Sciences, Tampere University, Tampere, Finland; Centre of Preventive Medicine and Digital Health (CPD), Medical Faculty Mannheim, University of Heidelberg, Mannheim, Germany; NIHR Policy Research Unit, Newcastle University, Newcastle upon Tyne, UK; The Unit of Social Research, Faculty of Social Sciences, Tampere University, Tampere, Finland

**Keywords:** behavioral insights, complex systems, behavioral public policy, semi-structured interview, expert interviews

## Abstract

Amidst the global momentum of behavioral insights (BI), there has been a shift from mostly nudge-based BI applications to systemic approaches. This is particularly pressing in public health, where interacting issues regularly produce unanticipated consequences. Regardless, little is known about adopting complex systems approaches in behavioral public policy. This study aims to capture current practices of international BI experts on the definition, application, drivers, and hindering factors in adopting complex systems approaches in public policy. Semi-structured individual expert interviews (*n* = 12) of international BI experts with extensive experience in educating, cooperating with, and/or advising public servants were analyzed with inductive content analysis. While the working definition of BI aligned with published definitions, experts varied in their descriptions of complex systems approaches and its significance for public policy, including socioecological aspects, systematic BI use across policy stages, recognizing intertwined behavior, and lack of ready-made solutions. They emphasized the importance of systems approaches, identifying drivers (e.g. need for a broader focus) and hindrances (e.g. pressure for quick results). Embracing complex systems in behavioral public policy provides a holistic perspective, extending beyond simple nudges, sometimes presumed as universally applicable. While complexity perspectives would align with policymakers’ worldview, applications require more work to tailor to local situations and to evaluate. Recognizing that, given their distinct expertise content, BI expertise can be quite different from complex systems expertise. The field would benefit from clear descriptions and specialized training for effective integration and advocacy for these approaches.

## Teaser

Behavioral insights experts identified several drivers and barriers to adopting complex systems perspective in behavioral public policy.

Hindrances for the use of complex systems approaches involve pressure for quick results, simple communication interventions being easier to implement, inconsistency in concept usage, and an oversimplified view of behavioral insights.

**Implications**

**Practice:** Public policy practitioners need to adopt a multifaceted understanding of behavioral phenomena, ensuring that systems approaches complement, and extend nudge-based strategies.

**Policy:** For an effective integration of systems approaches, policymakers must acknowledge the limitations of nudges and proactively address identified barriers, such as pressure for quick results, simple communications-based interventions, inconsistent use of behavioral insights concepts, and oversimplifying behavioral insights, to promote and institutionalize the use of complex systems approaches in public policy.

**Research:** As the field advances, future research efforts should prioritize the exploration of the drivers and barriers of applying systems approaches in public policy, ensuring that behavioral insights are firmly grounded in a rich understanding of the complex systems they operate within.

## Introduction

Behavioral insights (BI), defined as the application of behavioral economics and more broadly as the knowledge from behavioral and social sciences [1], is an important issue today in various parts of public policy. Behavioral science approaches have been increasingly used in public administration and policy during the last decade [2, 3], and many governments around the world have established “behavioural insights units” or behavioral science groups to improve governance with the use of behavioral science [4], as well as governmental institutions such as World Health Organization [5]. BI have been used all over the world across different policy levels and a large variety of policy areas, such as health, employment, environment, finance, and taxes [1]. For example, in recent years, the role of behavioral and social sciences in addressing the covid-19 pandemic was highlighted by institutional actors [6], and scientists alike [7]. The application of behavioral science to public policy often relies on dedicated expertise that is available through specialist groups within public administration, or through external support by university or company-based units. With their advice, evidence provision, and contributions, these units shape up to a good degree how BI in policy is realized [8–10].

Nudging is a one—and perhaps the most famous—way to use BI in policy, including promoting public health and healthy behaviors [11]. Nudging includes changing environments or altering the choices on offer so that more intuitive decision-making processes are employed and making, e.g., healthy choices are made easier, without preventing any options or giving vast financial inducements [12–14]. Nudges were initially adopted by many parties as the main behavioral science tools [15], and due to being a cost-effective and well-known way to use BI in policy, there might have been political and budget-related temptations to use mainly nudges [3]. These approaches have also aligned well with certain political approaches that had been dominant at the time of their increasing success [16].

While nudging added tools for governments to influence behavior change via other routes than simply regulation and legislation, the increasingly heavy focus on nudging in the early 2010s in, e.g., the UK was criticized [17], as many other behavior change intervention types and approaches exist [18], that may be more appropriate to address certain policy problems. Several frameworks exist that help systematically incorporate behavioral theory and evidence into practical interventions and policies [19, 20]. Indeed, it has been suggested that BI in policy should not be equated with nudges [21, 22] and that using BI in policy should move from nudging individual behaviors to more extensive approaches [3].

It has been suggested to move from nudge-based behavioral public policy (BPP) to advanced BPP where, instead of limited scope of problems, the scope and the scientific basis would be wider and the whole systems approach would be used [22]. BI are now often considered to encompass a wide range of behavioral science tools and not just nudging: For instance, BI have been suggested to be fully integrable with and able to inform other traditional forms of intervention (i.e. regulations, incentives, and information requirements)—and thus, BI may support a broad range of policy instruments [21]. One reason for the switch is also that it is not clear if an approach based on individual decision-making offers equity, or as a matter of fact increases inequalities [23].

The recent shift from mostly nudge-based applications of BI to broader approaches [15], has seen a more extensive incorporation of systems approaches [24]. However, for example in health promotion and policy, the suggestion to approach the problem from a complex systems perspective is not new albeit also has not been mainstream [25]. In complex systems approaches, human systems are referred to as *complex* instead of merely *complicated* [26, 27]. What this means is, that they are not simply sums of a large number of independent parts, but products of inseparable, dynamic interrelationships, which can change abruptly, and where *patterns* produced by the parts are not derivable from studying the parts in isolation. For example, consider how different water and steam are from each other, even though they are made of the same molecules: the difference arises from the (absence of) ties that hold the H_2_O molecules together. Given that connections between parts can be more meaningful than the components themselves, complex systems science “focuses on how the components within a system are related to one another,” and hence on universal features of systems across disciplinary boundaries [28].

In behavioral sciences, the most common applications have probably taken place in the area of health, as outlined in the UK Medical Research Council’s recently updated guidance for developing and evaluating complex interventions [29], as well as the abundance of calls to incorporate complex systems insights into public health [25, 30–33]. As an example, a review of organizational interventions to improve care of Type 2 diabetes showed that those interventions, which were aligned with principles of complex systems, were more effective than those that were not. These were interventions that took into account that organizations are interconnected systems, have learning, active agents, they self-organize, and co-evolve [32]. As an example, a community may be on average somewhat affected by an intervention to increase physical activity but revert to its baseline when the energy provided by the intervention fades. On the other hand, if the intervention manages to significantly affect a critical mass of people, even though the average is not drastically altered, the community may experience a shift in collective norms, which then start to exert “downward” influence over the individuals. This process can be described as an abrupt “phase shift” followed by crossing a tipping point, and has the potential to contribute to more lasting effects—or disastrous risks [34].

While nudge-inspired public policies can lead to success in realizing policy goals, many of the problems are so complex that such interventions are not sufficient. It has been argued that as the global arena grows more interconnected, it also increases in complexity [35]—as an implication, BI work needs to adapt to match the challenges posed by non-linear changes and unintended consequences of interventions [24]. Complex systems approaches to behavior change [34, 36] have been outlined to aid in this endeavor.

Against this backdrop, it becomes crucial to explore how experts in BI perceive and navigate this transition toward complexity. These experts play a pivotal role in integrating (behavioral) science into policy and their perspectives on the complex systems approach are instrumental in shaping the future of BPP. To date, however, the extent of the adoption of complex systems perspectives in this realm remains uncertain, and the viewpoints of these experts remain largely unexplored. Our study aims to fill this gap. By capturing the experiences and insights of behavioral experts, our study aims to illuminate the nuanced role of complexity in BPP, informing its future design and implementation.

Our research questions are as follows, (i) What kind of definitions or descriptions do experts give to BI?, (ii) How do the experts define or describe systems approaches in BPP?, and finally (iii) Is the change toward an increased role of systems approaches in public policy happening, and if so, what is driving it and hindering it?

## Methods

### Recruitment and data collection

The research data consists of semi-structured individual interviews (*n* = 12, six men and six women) of international experts with extensive expertise in educating, cooperating with, and/or advising public servants and who were based in Europe (*n* = 7), Australia (*n* = 2), North America (*n* = 1), and internationally operating organizations (*n* = 2). Purposeful and convenience sampling was used in data collection. The research group and a collaborator used their networks to identify potential participants working at BI units. The goal was to map BI teams that had been operating for several years, instead of only recently established ones, and to invite experienced experts, such as people in senior positions, from those groups. No assistants or people in junior positions were invited to participate. The first author and a research assistant contacted potential participants individually by email. Additional international experts working with behavioral advisory and training civil servants were identified by searching the Internet. In total, 21 email invitations were sent. Fifty-seven percent of the invited experts with a working email address agreed to participate. Six invitees did not answer the invitation, and three refused due the lack of time to participate at the time.

At the time of the interview, the participants worked at either national or regional BI units, international NGOs, or/and universities. The participants were experienced experts in leading or senior positions working around the BI topic area. Many participants worked in senior positions within the public sector in state government or ministries, or international NGO (*n* = 6), roles varying from head of some topic area, senior behavioral advisor to senior specialist. Some worked as project managers/coordinators (*n* = 4) or as scientist/lecturer at the university (*n* = 2) involved in practical BI work in public policy. The participants worked around, e.g., topics such as consumer protection and health. Eight were working as supervisors. Most of the interviewees had applied BI in one country only but some had worked in several countries. Altogether, participants had applied BI across Europe, the Middle East, North Africa, Southern Africa, Central Asia, Southern Asia, the Far East, Australia, North America, and South America (39 countries in total were named).

The first author and a trained research assistant conducted and recorded the interviews via Microsoft Teams during summer 2022 (see the interview guide in Supplementary File 1). The interviewers who worked as research assistants had 2 years of research experience, including conducting semi-structured interviews and data analysis. The interviews were conducted in English, and, on average, they lasted for 48 minutes (ranging from 39 to 57 minutes). Not all the participants were native English speakers. The recordings were transcribed by a trusted and regularly used external service provider which offers transcription and research services, and which was authorized by the university with framework agreement. The entire text corpus consisted of 75 797 words in total. Written information about the study and data management was provided to participants during recruitment. The main content was orally covered again at the start of the interview with additional information on the practical aspects of the interview. All provided an informed consent orally on the audiotape. The study protocol was reviewed by The Ethics Committee of the Tampere Region (Statement 86/2022).

### Analysis

The data were analyzed by the first author using qualitative content analysis [37] during spring 2023. This method aims to construct a condensed and general form description of the phenomenon under study [37]. As our aim was to study experts’ perspectives of BI in public policy, the association between participants’ experiences, meaning, and expressed language was assumed to be straightforward and unidirectional. Thus, we take a realist/essentialist epistemological standpoint, that assumes that the interview speech reflects experiences and reality of the interviewees. Semantic approach was used in the analysis, meaning that analysis focused on the manifest content of the experts’ recorded and transcribed words. The writer team discussed analyses during the whole analysis process in several regular meetings. The study team had considerable experience in using these approaches, also with policymakers, however, the first author conducting the analysis had no practical experience in BI in policy.

In the beginning of the analysis process, the first author read the transcripts and listened to the interview recordings several times to get an overall picture of the whole data. In addition, two members of the author group listened and/or read the transcripts to get familiar with the interview data to give their informed opinion about the coding.

Next, the transcripts were re-read now focusing on the answers given to the interview questions regarding BI and systems approaches and other parts of the interview that included speech about BI (e.g. *BI is about addressing behavioral biases*) or contained mentions about systems approaches or complexity (e.g. *we change the question to look more at the system*). The descriptions of BI and systems approaches were selected as suitable units of analysis. Important insights about the BI and systems definitions and the role of the systems approaches were identified, and the research questions were clarified based on these insights.

The inductive content analysis process contained open coding, grouping codes, creating categories, and lastly abstraction [37]. The preliminary coding, which included marking the data extractions and their condensed expressions, was conducted by the first author for the whole interview data focusing on the parts where BI and systems were talked, and the codes were discussed with the author team. Next, the codes were grouped by similar condensed expressions, and preliminary categories were created based on the coding. The final round of coding was conducted, and categories and subcategories were discussed with the author team. Finally, the first author modified categorizations and subcategories. The meaning of the categories (and their relationships) was interpreted during the final abstraction phase. Standard reporting guidelines for qualitative research were followed (see SRQR checklist in Supplementary Materials).

## Results

### The identified BI definitions in experts’ interview talk

As for the first research question regarding descriptions of the BI working definitions, we found four main categories, which are partially overlapping (Supplementary Table 1): (i) *Scientific basis* (subcategories Disciplines and Research), (ii) *Knowledge and understanding of human behavior* (subcategories Decision-making and biases, and Human aspect), (iii) *Methods* (subcategories Experimenting and Using evidence), and (iv) *Applying to policy* (subcategories Work and general applying, as well as Societal change and improving services). In defining BI, the common ground among all interviewees was that BI could be condensed to behavioral sciences and applying them to policy. The most common descriptions included mentions about different behavioral disciplines and using those insights to real issues.

#### Category 1: Scientific basis

Most of the experts defined BI by referring to behavioral sciences and several disciplines, such as social psychology, and behavioral economics. While also disciplines such as sociology and anthropology were named, one participant was critical of whether they should be included in behavioral sciences.

P9: “I would define it as bringing the latest insights from social psychology, anthropology, and sociology, and related topics, in the design and implementation in public policy programmes.”

Scientific basis of BI was also referred to as theory use and as overall research, not naming any specific disciplines. BI was defined “as the knowledge and the research of behavior and its change in its very many forms” (P11).

#### Category 2: Knowledge and understanding of human behavior

Although the scientific basis dominated the definitions of BI, a couple of experts presented BI with an overall idea of understanding *human decision-making and biases*:

P4: “I think the original spirit of behavioural insights is to understand that people [--] aren’t, most of the time, [willingly] following the rules or [making] optimal decisions.”

Also, *‘human aspect’* and understanding the reality of people were mentioned:

P2: “[--] the way I understand it is that in our work, at least or the what we do in the public sector, whenever we develop services or programmes, or strategies, then we should take into account the human aspect, and the human aspect on a very, sort of a, first of all on a holistic level of, everyday daily life, of people, so the whole general context of where people are operating [--]”

#### Category 3: Methods

*Experimenting* and *using evidence* was mentioned when defining BI, and conducting experiments was mentioned also in other parts of the interview. Therefore, experimenting and using evidence (gained via using research methods) seems to be central for BI in public policy:

P4: “So a brief definition of behavioural insights that we use in our team is the application of social and behavioural sciences to improve services, programs, and social policy. So what that means is that we get evidence from the behavioural sciences about what works to change behaviour [--]”

#### Category 4: Applying to policy

BI was conceptualized with descriptions about experts’ own work and more generally by mentioning applied work and applications. Making societal change happen and improving services were mentioned.

Still, there were some concerns about BI not being so beneficial in higher levels of public policy and some were pondering if ministerial level is the right level for applying BI. This was rationalized by stating that there is more distance to end users in higher policy levels, while, e.g., a “step below the ministrial level” (P12), BI could be applied better to practical work and it would make a bigger difference.

### Descriptions of the systems and complexity approaches

With regard to the second research question, interviewees’ descriptions of the systems and complexity approaches were categorized in three main categories (Supplementary Table 2): (i) *Socioecological view* (subcategories Move from individuals to groups and organizations and Considering structural and cultural aspects), (ii) *Systematic way of using BI in different stages of policy cycle*, and (iii) *Complexity approach* (subcategories Intertwined phenomena, No copy-paste solutions, and Tools of systems approach).

#### Category 1: Socioecological view

Within socioecological view, the most central view was that systems approaches seem to mean shifting from simple and individualistic focus to more complicated and context sensitive ways of using BI. For many experts, (the shift from nudges to) systems approaches seems to mean a shift from individuals to focusing on a broader context. The experts described *focusing more on groups, organizations*, and taking meso and macro levels into account. With these views, they acknowledged there being multiple types of influences to consider when moving to systems approaches, although the idea on its own is not strictly speaking fully congruent to complex systems perspectives.

Also *considering structural and cultural aspects*, such as factors and barriers were described when talking about systems approaches and were also described through experts’ own work. For example, equity and diversity dimensions were mentioned:

P4: “So for example with the current work on improving women and girls’ recruitment. The biggest factor is actually just gender norms and gender discrimination, and while that’s made up of behaviours, which can be addressed through behavioural measures. It’s actually a broader structural problem, obviously so. In that case for example, we’re really clear about what we can and kind of shift in terms of behaviour.”

#### Category 2: Systematic way of using BI in different stages of policy cycle

A few experts described using BI systematically in all levels of policy cycle, not only in the end point when thinking about solutions. This was seen as challenging but worth doing.

P10: “You know we’re thinking more about using behavioural insights during design rather than just to kind of fix problems at the end point. I think that’s another big challenge for us, as we get more, more respected as a field.”

#### Category 3: Complexity approach

The descriptions in this category resemble characteristics that are familiar to more traditional conceptualization of complex systems approaches. Only some experts were mentioning ideas of *intertwined phenomena* and different parts of the systems affecting other parts of systems:

P10: “I think we’ve gone from focusing on kind of really narrowly defined problems to now pushing at that barrier of like where can we make, how can we look at this from a systems perspective to look at where we can have the most impact and also how what we do in one part of the system impacts the other parts of the system.”

One interviewee was proposing a different conceptualization of systems approach, and intertwined phenomena was referred to long-lasting patterns with structural dynamics:

P4: “The second generation nudges, which I think I sort of heard behind your question, is more about complex behaviours. Like quite a lot of, all of, exclusively all of my work. So recidivism, which is a long-standing pattern, which is connected to structural dynamics.”

In addition, some experts mentioned that there are *no “copy-paste solutions,”* or common solutions, to all different situations when using BI:

P6: “I feel like that in more recent applications of behavioural insights, there is a trend that we’re seeing where more systemic factors are being taken into account and, we’re moving from a rather simplistic or, catalogue solution oriented perception of behavioural insights into the integration of much more complex systemic factors.”

Statements about having to test and not using the solutions from other contexts were also mentioned at a general level by other interviewees. Also, the idea of complexity and having no copy-paste solutions were seen when a couple of the experts talked about unanticipated factors and consequences that can emerge regarding behavior.

Lastly, in experts’ descriptions about their work, usage of common *tools of systems approach*, such as systems mapping and analyses, was mentioned:

P11: “[--] when we think about the behaviour change in general that we also think about the systems, but also as a method, as we try to sort of comprehend the depth and the effects of a particular problem or a particular policy that we work with, and we have done some system analyses, when we have worked with the civil servants.”

### The role of the systems and complexity approaches

Regarding the role of complex systems approaches for BPP, there were three types of stances among interviewees. First, most of the experts stated that systems approaches have a role, even “a big role” (P4), and the shift from nudges to systems approaches is important. Experts justified the role of systems approaches either with general importance in public policy or with their own use of systems approaches in their work. Interviewees used temporal narratives to describe the shift from the past (nudges) to the current situation (systems approaches), e.g., “five to eight years ago that all the craze was about nudging (P2)” and “there’s been kind of broadening of focus from the early days where nudges were extremely popular and the focus was very much on individuals (P6).” Still some thought that systems approaches are not used as comprehensively as they could and there is room for improvement. Secondly, only a couple of the interviewees saw that the systems approaches does not play a role in public policy, one even stated it being a marginal approach, being dependent on the people working within public policy or the people advising policymakers. Thirdly, some were ambiguous or unsure about the role. Overall, the shift to systems approach is seen as very positive and preferable in the context of public policy.

### The drivers and barriers of the systems and complexity approaches

Finally, we analyzed two drivers and four hindering factors. Drivers included (i) *Need for broader focus or a more holistic picture*, and (ii) *Experience leading to expertise to use systems approaches*. Hindering factors were categorized as (i) *Advisory settings and pressure for straightforward or quick results*, (ii) *Simple communication interventions are easier*, (iii) *Inconsistency in concepts and approaches*, and (iv) *Simplistic views of BI* (Supplementary Table 3).

#### Driver 1: Need for broader focus or a more holistic picture

The need for broader focus appeared to be a driving force to use systems approaches. The desire to broaden the scope from individuals to organizations and understanding the complex nature of human behavior seemed to guide promoting systems approach. In addition, limitations of nudge’s potential were also mentioned as nudges were seen as only one tool among others and they were not seen as sufficient tools to broaden the focus. Still, nudges and simpler interventions seemed to have created requests for BI, and in that sense also for systems approaches. When BI teams have been mandated with easier solutions and demand for BI has been created, they could more easily use more complex approaches.

P2: “[we’ve also moved] within that direction, that we look at the systems as a whole, we see how they interplay [--] what are the seen factors what are the unseen factors and so forth, that we should take into account because otherwise our solutions will not work, if we don’t [involve] such understandings.”

#### Driver 2: Experience leading to expertise to use systems approaches

Expertise or experience to use systems approaches are important drivers of using systems approaches. As BI teams have had more knowledge and experiences doing basic BI work, they can more easily use more holistic approaches in the future. In addition to getting more experience, BI team size was also mentioned as either a driver (suitable amount of experienced team members), and hindering factor (too little team size) which may have also influenced the results of expertise. Conversely, lack of expertise was also mentioned once as a barrier of using systems approaches.

P1: “Then, when you are creating knowledge in the team, and the team [--] is bigger now. [You] start to think about consumers and the problem [--] in a more systematic way, I will say. Trying to match different biases, the environment in which consumers take decisions, so, we are working on that area now but of course, it was a process of learning while doing [--]”

#### Hinderer 1: Advisory settings and pressure for straightforward or quick results

As hindering factors of increased role of systems approaches in public policy, interviewees mentioned that conditions of advising often put pressure for straightforward and quick results and simplified advice where it is not always possible to consider complex systems. The pressure was not always a direct requirement from policymakers but moreover relating to the characteristics of policy-making processes and time resources allocated to advising. The advisory sessions can be brief (e.g. short meetings) or in the written format (e.g. short texts) without a possibility to verbally explain the advisory contents which is forcing BI experts to start with simple advice, and makes it hard to know to what extension systems approaches are used:

P8: “If you look at advising policy makers, it's important to realise that most of these advisory moments can be written materials [--]. And you can also sometimes be around the table with the policy makers but these are then often quite brief. [--] So for me it’s quite difficult to say to what degree policy makers approach behavioural issues from a complex dynamic system approach.”

Due to the demand, nudges and so to say low-hanging fruits (easy interventions) were seen as a good start and a way to justify the value of using BI at all. Thus, one mentioned that with systems approaches there is possibility to get more impact.

#### Hinderer 2: Simple communication interventions are easier

Simple communications-based interventions were seen as easier to carry out and thus were especially used in the early days of BI teams. Being cost-effective and quick were also mentioned to urge the use of simple interventions. Overall, several interviewees had been conducting communication-based interventions, such as SMS messages and redesigning documents. Communication-based interventions do not only mean not using systems approaches but it was mentioned that “of course to make better communication [--] more systemic approach is very important, but also challenging” (P7).

#### Hinderer 3: Inconsistency in concepts and approaches

In addition, inconsistency and discrepancy in concepts and approaches makes it harder to apply BI in policy. One interviewee mentioned that in some countries there is “a mess of different approaches,” when assessing systems approaches’ role.

#### Hinderer 4: Simplistic views of BI

There were also interviewees that saw that there are simplistic views of BI which make it harder either to assess the role of systems approaches or to use them. At times “trendy” or more popularized views of BI can stand out among public servants and lead to think more about individual behaviors than the system, or BI can be seen as a synonym to nudges or consisting only of concepts such as System 1 and System 2. Still, this was not limited to public servants, but one interviewee saw that other people working within the BI area could also be guilty of it, as all of the BI experts have not taken traditional behavioral science training and people outside behavior science field could “water out the concept of behavioural insights” (P5).

Still, there are mixed opinions if public servants are more interested in systems approaches. An expert said that in general civil servants need to be guided to think about systems approaches instead of focusing on individuals as they do not think about systems approaches naturally. An interviewee said that “there is not that direct call from higher level policy makers to come up with a certain approach” (P12), but certain approaches are introduced to the policymakers by BI experts involved in the current times. In turn, one interviewee said that public servants often ask if BI is only nudges or is there something more.

## Discussion

This study examined the perspectives and insights of international behavioral experts on the definition, and role of complex systems approaches in BPP. The results draw a comprehensive picture of the utilization of BI and systems approaches in public administration from the perspective of experts advising on BI in public policy. The results show that experts had a shared view of BI as the use of scientific basis, knowledge, and methods from behavioral sciences to apply in public policy, which is in line with common definitions of BI [1, 21]. The descriptions of systems approaches included using BI systematically in different stages of the policy cycle, but there were also descriptions that reflected familiarity with systems thinking. For example—and as could be expected—participants described the socioecological view of moving the scope from individuals to groups and organizations and once again in structures and culture. Systems approaches were also described in terms more aligned with complex systems approaches, involving dynamic interconnected parts. In such scenarios, using tools of systems approaches played a role when it was seen that there are no copy-paste solutions and there might be behaviors’ unanticipated outcomes.

In addition, the results reflect on the important role of systems approach and the need to move BI from isolated individuals toward viewing them as agents interacting in a complex system. Most experts saw that there is a shift from nudges to systems approaches, especially in more advanced use of BI, and that was seen as an important development. Still, there were also some ponderings whether the full potential of using systems approaches in public policy had been reached, and some stated that BI in public policy is moving toward systems but is not there yet. Views of systems approaches having no role in BPP were also presented.

Overall, the context of public policy might not optimally support the uptake of systems approaches. Some experts described how there can be pressure to give simple solutions and brief advice even though the behavioral issues can be complex. There can be limited time and resources, and brief advisory sessions or short written materials do not encourage the use of systems thinking; demand is for simple advice and simple answers to complex issues. Although many experts stated that systems approaches have an important role in public policy or at least it is a welcomed movement, the experts in many cases described work around simple and fast communication-based interventions, i.e., designing different kinds of communication documents and evaluating them in letter- or SMS-based trials.

It seemed that there was both supply and demand for communication-based interventions—one interviewee even stated that redesigning documents was their team’s main focus. Such behavioral interventions were seen as cost-effective and meaningful, even as a default in public policy. Also, small demonstrations of results were seen to justify the value of BI and get more budget and employees in the future. There has been a justified need to use more simpler applications of behavioral science, so called “low-hanging fruits,” especially in the beginning of BI teams’ work, but BI are more than applicable to be used in complex problems [2].

Yet, this does not mean that complex systems would always require complex interventions. In fact, sometimes the best solution could benefit from numerous micro-interventions that could enable the system to change, and perhaps, at some point, lay out the necessary path for the system to slip into a new state. So, while it is unlikely that clarifying communications would solve extremely complex problems by themselves, it does not mean they are at odds with systems approaches. It should also be noted that complex systems science is a highly heterogeneous field. In this sense, the interviews also reflect this meta-complexity, where considerable progress has been made in different areas without even a single, consensus-based definition of “complexity.”

In addition, there were notes about simplistic views of BI which are, on the other hand, understandable since the idea of BI has been “sold” as the key solution in public policy, despite that there is more to behavioral science. Therefore, behavioral scientists, including those using complexity approaches, should try to make evidence, especially the complex systems evidence, understandable. Still, one other barrier to advancing the use of complex systems approaches might be that those with experience in policy advising might be more comfortable in their traditional BI expertise and less knowledgeable about complex systems.

Embracing complex systems approaches in BPP has the potential to significantly improve our ability to navigate modern challenges from misinformation to the existential threats of pandemics and climate change. This could mean shifting emphasis away from centralized, top-down communication of blanket solutions, toward coordinated portfolios of parallel, e.g., community-led experiments [27]. Starting from distinguishing complex decision-making contexts from those amenable to large-scale solutions [26–28], this lens can inform interventions that are responsive to local contexts and resilient to unintended consequences. Ultimately, complementing traditional BI with systems perspectives enables us to better understand the dynamic, interconnected nature of real-world public health challenges. This can lead to the identification of system-changing interventions, and public empowerment to create choice environments that generate sustainable, improvements in health and well-being.

## Limitations and reflections

This study studied definitions of BI and complex systems approaches and its role in public policy. Although the study succeeded in illustrating the experts view of systems approaches’ current role, some limitations are present. It should be noted that this represents a single-sided reflection of a dyadic process and misses the perspective of policymakers who make use of BI in their work. In the interview, systems approaches were to discuss by referring to a recent trend in BI as a move from a sole focus on nudges to more systemic perspectives. The interview question formations might have influenced the answers’ emphasis on nudges as there was considerable amount of speech about nudges. All the categories of systems approaches’ definition had in common that systems approach was seen to be related to a shift from a perspective focused on individuals to a broader one and a shift from a simple to a more complex one and systems approaches were compared to nudges. By asking experts especially to define systems approaches, they could have had more elaborate descriptions of them. Also, after experts commented on the role of systems approaches, no additional questions about the topic were presented which may have left some things unsaid and resulted in the text corpus regarding the systems approaches being quite short. There might have been some additional speech about systems approaches in other parts of the interview, but the analyst was careful not to include unsure parts in analysis to avoid too vast interpretations. In addition, due to limitations in sample size, some initial subcategories could not be included in the final results. Also, it might have been insightful to study if the statements regarding the role of the systems approaches depended on the interviewees’ backgrounds. Due to limitations in sample size and details of the experts’ backgrounds, the current data will not shed light on this topic.

At this point of time, limitations in sample size might be due to difficulty to recruit experienced BI experts in leading positions as there are a limited number of organizations and people applying BI in policy globally and the number of potentially includable participants is low to begin with. Still, similar amount of interviews has generally been considered sufficient [38] and due to the saturation of insights, data saturation seems to have been reached. In the analysis process, many codings seemed to describe same phenomena and categories could be found from many parts of the data. In addition, it is likely that a couple of more interviews might not have added to the results.

## Conclusions

This study contributes to improving our understanding about the role of complex systems approaches in BPP from the perspective of international BI experts. While our results show that the experts mostly see systems approaches having an important role in BPP, their accounts about systems approaches were heterogeneous and the descriptions of systems approaches adaptations differentiated. This might make it hard to apply complex systems approaches in public policy in a broad sense. Thus, while systems approaches were seen as welcomed development, there is a need for more elaborated consensus of what systems approaches mean in the context of public policy. There are also some barriers in the context of public policy that make it difficult to accomplish the full potential of BI. While applying a complex systems approach hence requires greater investment in training, participatory practices, and adaptability, it can be essential for confronting the contemporary challenges arising from increased global interconnectedness. Public policy would benefit from developing practical tools and guidelines for integrating behavioral and systems insights into policy-making processes.

## Supplementary Material

ibae024_suppl_Supplementary_Files

## Data Availability

De-identified data from this study are not available in a public archive.

## Welfare of Animals

This article does not contain any studies with animals performed by any of the authors.

## Transparency Statement

*Study Registration*: This qualitative study was not formally registered. *Analytic Plan Pre-Registration*: The analysis plan was not formally pre-registered. *Analytic Code Availability*: There is no analytic code associated with this study. *Materials Availability*: All data collection materials (participant information sheet, privacy notice, interview guide, and instructions to the interviewer) used to conduct the study are available in a public archive: https://osf.io/6j2wy/ Interview guide is also shared as Supplementary File 1.
