# Supplementary material for: How do behavioral public policy experts see the role of complex systems perspectives? An expert interview study
Source: Transl Behav Med. 2024 May 22;14(7):417–25. doi: 10.1093/tbm/ibae024 (PMC11208291; doi:10.1093/tbm/ibae024)
Supplement: ibae024_suppl_Supplementary_Files [file ibae024_suppl_supplementary_files.pdf]

How do behavioural public policy experts see the role of complex systems perspectives? An expert interview study

SUPPLEMENTARY MATERIAL

Supplementary file 1

Interview guide:

[Underlined questions are meant to be asked from all interviewees (except when the interviewer's notes indicate otherwise).]

Background information

The results of this interview can be reported in a scientific publication or other report, so I'll ask some questions about your background. These are not published in an identifiable form or mentioned together with a direct citation in a way that could make identifying you possible. For example, the gender distribution might be reported in summary form.

- What is your job title or profession?
- And what is your organisation and sector?
- Do you work as a supervisor?
- And what is your gender?
- Your nationality?
- In which roles and in which countries have you carried out behavioural insights related to consultancy, advisory, training or promotion?
    - *If the interviewee tells about experiences outside the scope of this study:* Thank you so much - You clearly have such vast experience, I should highlight that in this study we are interested especially in the more recent experiences, like in the last 10 years or so.

Definitions:

1. People can understand behavioural insights in many ways, can you tell me how you would define behavioural insights and how your work is related to it?
    a. If need for more speech: What terminology do you use in this context?
    b. Additional questions, if not enough speech:
        i. What kind of tasks are included in your work?
        ii. What are the goals of your work?
    c. *If hasn't said yet:*
        i. In what kind of ways have you implemented or introduced behavioural insights perspective to governments? (e.g., trainings of public servants, and/or consultation in projects, and/or what?)
            - *If a person has not explicated whether they've done behavioural science advisory work and/or trainings:* Just to be sure I understood correctly; have you delivered trainings to public servants, and have you done advisory work or consultations?
            - *"Not myself, but my unit has done": How have you been involved in the trainings/ your unit's advisory work?*

- *If has experience from both advising, and trainings:*
    a. As we have only one hour for this interview, we will concentrate more on either advisory work or trainings you've conducted. Which one do you have more experience of, which one would you prefer to talk more about? We will have that one in our focus.

TOPIC 1: Own work and use of behavioural knowledge within public sector

So there exist many different definitions of "behavioural insights" (BI) to date, for example by OECD and European Commission. In this interview, we are interested in knowing your opinions and experiences regarding behavioural public policy *widely defined*, for example the use of behavioural science theories, for example from psychology, and the use of behavioural evidence, and behavioural science methods in policy.

2. One recent trend in BI has been the move from a sole focus on nudges, to more systemic perspectives. What kind of role, if any, do systems or complexity approaches play in your view of behavioural public policy (/this sphere)?
3. In your opinion, are there problems with terminology in this area currently, that hinder its usage?
4. What are the most asked questions about behavioural phenomena when working with public servants?
    a. If need for more speech:
        i. For example, what kind of behavioural phenomena puzzle public servants?
        ii. What needs to be explained often?

TOPIC 2: Barriers and facilitators

5. When introducing the use of behavioural insights to governments, what have been the most usual **misunderstandings** among public servants or policy makers? Such misunderstandings could relate to behavioural phenomena, methods or theories.
    a. For example, some lay beliefs may include that providing information is sufficient to change behaviour.
6. What have been the most usual problems or barriers when introducing the use of behavioural insights to governments, in your experience?
7. On the other hand, what have been successful ways of introducing behavioural insights, in your experience?
    a. Examples of success stories?
8. *This doesn't have to be asked, if already covered when answering to Q7.* Overall, what kind of things have been the helpful factors, or facilitators, in introducing the use of BI to governments?
9. If you could do your work all over again (in introducing behavioural insights approach to public policy formulation), what would you do differently now?

TOPIC 3: Training public servants in behavioural science approaches: experiences and lessons learnt - *Only, if has conducted trainings*

10. Now we will move on to behaviour insights (or BI) trainings. With "training", we mean any activity in bringing about BI knowledge and skills to the public servants: For example, talks,

lecture series, workshops, courses and so on… What kind of trainings, if any, have you organised, that aim to teach the public servants or policy makers to use BI themselves?

   a. What has been the target group? What kind of work they do?
   b. What were the learning objectives in the training?

11. What behavioural **frameworks or tools** were taught (in trainings)?
    a. Additional questions, if need for more speech:
       i. What worked well in teaching frameworks or tools?
       ii. What were the things that participants understood well?
       iii. What did they not understand or struggled to understand?
12. In many governments, introducing behavioural insights has also included advancing the use of **empirical research methods** to gain insight into behavioural phenomena and to design interventions or policies. In and after the trainings, what kind of research methods have the public servants found to be the most useful?
13. What are some major learnings you've discovered upon delivering BI trainings?
    a. Is there something you would do differently now, if you were conducting trainings?
14. How did you organise the trainings?
    a. What was the format (e.g., video lectures, live lectures with discussions, or what)?
    b. What was the duration of the training?
15. Did you gather any feedback? If yes, what kind of feedback did you get?
16. *If there's time, ask from all participants, who have conducted trainings.* In your opinion, based on all your experience, what would you say are the important aspects to take into account in training public servants to use BI themselves? In terms of learning objectives, teaching methods, training format, duration, or any other aspects.
    a. If you have gathered any feedback, what kind of feedback did you get?

TOPIC 4: Behavioural science advisory work - *Only, if has experience from advising*

And then moving on to advisory work.

17. What have been the most important goals in advising public servants on behavioural science issues?
18. To whom and how has the advisory been provided? What other actors have been involved in the co-operation and how?
19. What kind of knowledge, information or evidence has been used to inform the advisory work? How has it been produced?
20. What kind of effects has the work had, and how has it been evaluated?
21. Can you give an example of advisory work that has been successful? (You can think in terms of success to the advisees, or you as the advisor.)
22. What has been unsuccessful (in the advisory work)?
23. Which learnings about advisory work will make it easier in the future?
    a. *If the question feels too broad/difficult:* Could you tell an example of useful feedback you've received?

FINAL QUESTIONS IF WE HAVE TIME:

Now, I have some more general questions as we have more time.

24. Sometimes people have criticised BI to be unethical due to its "manipulative nature", as some people perceive it. What kind of experiences might you have of this, if any?
    a. If need for more speech: Have you had pushback related to perceiving BI as manipulation?
25. What other ethical issues have been raised in your experience?

26. It is commonly advised to involve the targets of interventions in the planning process. How have you incorporated co-production aspects in behavioural insights planning and their implementation?
    a. If need for more speech: What are your thoughts on advancing a sense of partnership between state, individuals and communities in the policy process?

Now to the final question… We have come to the end of the interview…
Is there something you'd like to add, before we end this interview?

Ask only, if the interviewee gave consent to archiving the data in the beginning of the interview: I will check at this point, so you still agree to archiving the pseudonymised interview transcript at the Finnish Social Science Data Archive?

Supplementary table 1

RQ1: What kind of definitions or descriptions do experts give to BI?

Note: Filler words have been removed and original quotes have been shortened.

Main category	Subcategory	Condensed extract
C1. Scientific basis	Disciplines	P7: “[--] using insights from the behavioural sciences, which is a very broad definition but at least it also includes social psychology, behaviour economics, and some other related social sciences [--]”
	Research	P12: “[--] in behavioural insights we combine research done for instance in economics, social psychology, in judgement and decision-making, in sociology and so on.”
C2. Knowledge and understanding of human behaviour	Decision making and biases	P1: “I will define behavioural insights as the branch of knowledge, [which studies] systematic biases in people’s decisions. Since we are doing studies and enforcing consumer law, we are permanently worried about how consumers are taking decisions in their daily life now.”
	Human aspect	P10: “I think how, the kind of operational definition we have of behavioural insights is using what we know about human behaviour whether that is from research, whether it’s from talking to people, from the work we do to understand what people do and how they think [--]”
C3. Methods	Experimenting	P6: “For example if you have a body of knowledge or experiments that are done in psychology the results of that science can be summarised into concepts that can then be used either to do applied work in policy or applied work for example in more business solutions.”
	Using evidence	P3: “Now behavioural insights belong to that idea of using evidence.”
C4. Applying to policy	Work and general applying	P6: “To me behavioural insights is basically the translation of findings from behavioural sciences into knowledge and operational insights that we can mobilise for more applied work. [--] In my case I use behavioural insights for policy, but more broadly speaking I think behavioural insights can be used for various applications.”
	Societal change and improving services	P10: “[--] using all of that information to then design better public services, to design services that work with how people think instead of against how people think.”

Supplementary table 2

RQ2: How do the experts define or describe systems approaches in behavioural public policy?

Note: Filler words have been removed and original quotes have been shortened.

Main category	Subcategory	Condensed extract
C1. Socioecological view	Move from individuals to groups and organisations	P9: "So that's kind of how we've seen the application of behavioural science, a lot more than just nudging for example and tweaking for the individual, we also look into tweaking the system and tweaking the group like influencing the system, but also influencing the groups."
	Considering structural and cultural aspects	P2: "[--] what we also always aim for in these analysis and synthesis phases, is trying to get the public servants understand what are the underlying social structures, the social forces kind of or cultural factors that play a role in many issues [--]"
C2. Systematic way of using BI in different stages of policy cycle		P7: "I think since, well, say, the last four years or so, we also tried to focus more on applying behavioural insights in policy development. And well, that is a good move 'cause in the end also we have more impact there, 'cause when you're only tweaking communication at the end the impact is less. [--]"
C3. Complexity approach	Intertwined phenomena	P2: "[--] we've also very, directly sort of moved within that context, or within that direction, that we look at the systems as a whole, we see how they interplay how they, what are the factors, underlying factors, what are the seen factors what are the unseen factors and so forth [--].
	No copy-paste solutions	P10: "[--] there's a misconception that if you lift something from somewhere else or design something in a thoughtful way that it's going to be impactful so that's a misconception we're often challenging that you actually need to measure and check if it's doing what you think it's going to do or if it's having an impact on different parts of the system that you weren't anticipating."

	Tools of systems approach	P2: “Our own approach I'd say is more also, of the systemic and the way that I've described how we have built up the programme and how we do the preliminary field work and the in depth interviews, is a lot about, this analysis and synthesis their goal is to show not really how the individual directly uses just you know, your single service or the touchpoint point of a single service, but to put them the service in a more holistic complex, to put it in a more social very often socioeconomic context [--]”

Supplementary table 3

RQ3: Is the change toward an increased role of systems approaches in PP happening, and if so, what is driving it and hindering it?

Note: Filler words have been removed and original quotes have been shortened.

Main category	Subcategory (drivers and hindering factors)	Condensed extract
Drivers	D1. Need for broader focus or a more holistic picture	P6: "[--] in more recent applications of behavioural insights, there is a trend that we're seeing where more systemic factors are being taken into account and we're moving from a, from a rather simplistic or catalogue solution oriented perception of behavioural insights into the integration of much more complex systemic factors."
	D2. Experience leading to expertise to use systems approaches	P10: "I've seen over the years that the ways that we've been able to use behavioural science and apply behavioural science have gotten more and more complex and rigorous which is great. [--] I don't feel like we've really mastered it, yet, but definitely I think there's a trajectory to think about that, more."
Hindering factors	H1. Advisory settings and pressure for straightforward or quick results	P9: "[--] people lose their attention so, you know their attention span is very small, so [--] sometimes you wanna be able to demonstrate incremental smaller, results to be able to show them the value of behavioural science, versus just conducting you know behavioural diagnostics."
	H2. Simple communication interventions are easier	P7: "[--] I think it's easier also so that you get to start with, where you have large scale groups of citizens and just change some communication there."
	H3. Inconsistency in concepts and approaches	P4: "[--] I don't think it's true to say that we've moved from nudges to structural and I think there's a lot of slippage in the way people use term nudge, just as as contention between what's a nudge and what's a boost and how can we measure the difference between them and."
	H4. Simplistic views of BI	P12: "[--] when you start discussing about nudging people, those civil servants very often forget how nudging is defined. And they very easily start calling pretty much everything as nudging, so.."
